# Editorial: Image-based computational approaches for personalized cardiovascular medicine: improving clinical applicability and reliability through medical imaging and experimental data

**DOI:** 10.3389/fmedt.2023.1222837

**Published:** 2023-06-23

**Authors:** Selene Pirola, Amirhossein Arzani, Claudio Chiastra, Francesco Sturla

**Affiliations:** ^1^ Department of Biomechanical Engineering, Delft University of Technology, Delft, Netherlands; ^2^ Department of Mechanical Engineering and Scientific Computing and Imaging Institute, The University of Utah, Salt Lake City, UT, United States; ^3^ PoliTo^BIO^Med Lab, Department of Mechanical and Aerospace Engineering, Politecnico di Torino, Torino, Italy; ^4^ 3D and Computer Simulation Laboratory, IRCCS Policlinico San Donato, San Donato Milanese, Italy; ^5^ Department of Electronics, Information and Bioengineering, Politecnico di Milano, Milano, Italy

**Keywords:** computational modeling, cardiovascular biomechanics, patient-specific models, surgical planning, diagnosis, cardiovascular imaging

**Editorial on the Research Topic** Image-based computational approaches for personalized cardiovascular medicine: improving clinical applicability and reliability through medical imaging and experimental data

Computational analysis is frequently integrated with medical imaging to develop novel and personalized solutions for the diagnosis, prognosis, and treatment of cardiovascular disease. Despite the significant increase in computational capabilities and the emergence of novel technologies that automate and accelerate workflows, the personalization of cardiovascular computational modeling remains a challenge with several unresolved issues. Multimodality cardiovascular imaging, including echocardiography, cardiac magnetic resonance (CMR), and cardiac computed tomography (CT), has significantly evolved in recent decades, enabling accurate and three-dimensional (3D) representation of cardiovascular anatomy. Furthermore, to account for the complexity of cardiovascular anatomy and function, mechanical properties of cardiovascular tissues and model boundary conditions should be prescribed on a patient-specific basis. However, obtaining these data directly from *in vivo* imaging can be challenging and may require additional imaging and a dedicated framework for data elaboration. In particular, model assumptions and simplifications may be necessary to avoid the use of invasive diagnostic imaging or to reduce the computational burden of numerical simulations.

The studies collected in this Research Topic address several key issues related to patient-specific cardiovascular modeling, with a specific focus on several aspects of the workflow limiting the clinical translation of computational analysis.

Two works in the present article collection focus on the segmentation and reconstruction of patient-specific geometries of blood vessels from clinical imaging. A trained deep learning-based approach was applied by Abdolmanafi et al. to achieve a fully automated segmentation of abdominal aortic aneurysm (AAA) tissues from CT, including arterial wall and lumen, intraluminal thrombus (ILT), and calcification. The automated segmentation showed very good agreement with the manual ground-truth segmentation, thus expediting the segmentation process and reducing inter- and intra-operator variability. An automated and user-friendly approach was proposed by Warren et al. to generate patient-specific 3D models of atherosclerotic coronary arteries by combining virtual histology-intravascular ultrasound (VH-IVUS) and angiography data. Leveraging an automated meshing algorithm for volumetric reconstruction of the arterial network, the program enables finite element (FE) analysis of stresses and strains across the arterial wall to facilitate the biomechanical assessment of atherosclerotic plaque stability. Notably, mechanical properties can be assigned to arterial tissues according to their specific VH-IVUS color-coded value.

Computational advances in cardiovascular biomechanics are possible by combining imaging with clinical data. Fruelund et al. proposed a novel non-invasive electrocardiographic imaging (ECGi) method based on a 12-lead inverse ECG algorithm that accurately reconstructed a 3D electrical ventricular activation map during right ventricle (RV) pacing and effectively identified the initial site of activation in relation to ventricular anatomy. When validated against the true CT-based RV pacing site, the reconstructed ventricular activation model could extend the clinical applicability of 12-lead ECG-based methods and overcome more complex ECGi methods requiring a dense array of electrodes. A new approach was proposed by Celi et al. to estimate the local distribution of aortic stiffness by combining *in vivo* dynamic knowledge of arterial morphology with clinical records of systemic pressure. To achieve this, a novel mesh-morphing approach based on radial basis function interpolation was applied to ECG-gated aortic CT images to map the aortic luminal surface over the cardiac cycle. A novel methodology to characterize the mechanical properties of AAA was proposed by Jansen et al. using non-ECG-gated freehand two-dimensional (2D) ultrasound imaging: a dedicated framework was established to semi-automatically segment and register probe-tracked images of the aorta at different phases of the cardiac cycle. Local aortic compliance and distensibility were subsequently computed using measured brachial pulse pressure values.

The promising results of the inverse modeling approaches developed in the last two studies and their application to real patient-specific cases suggest that the availability of time-resolved *in vivo* imaging can yield additional quantitative parameters of vessel wall biomechanics beyond 3D model geometry, such as regional characterization of deformation and stiffness. Using four-dimensional (4D) CT image data, Peng et al. showed that incorporating the aortic wall movement into the CFD analysis of AAA does not considerably alter the overall aortic flow field and wall shear stress distribution, as predicted by assuming rigid walls. However, it reveals greater blood stagnation in AAA, which may trigger ILT formation. Therefore, dedicated processing of *in vivo* images can help to configure boundary conditions that are relevant to the clinical goals of patient-specific CFD analysis. Using CFD analysis, Canè et al. examined how the interplay between left ventricular (LV) wall torsion and vortex guidance by the mitral valve (MV) affects intraventricular hemodynamics by combining patient-specific 3D models of the beating LV endocardium and patient-inspired MV leaflet kinematics through overset meshes. LV torsion significantly impacted the evaluation of blood stasis and residence time, and MV enhanced the inlet jet propagation toward the LV apex and apical washout.

Computational modeling can also be used to reproduce pathological cardiovascular conditions and investigate the severity of disease and the mechanisms of disease progression. In this context, Jafarinia et al. optimized an existing phenomenological shear-driven thrombosis model to predict thrombus formation in the false lumen (FL) of type B aortic dissection (TBAD) using a limited number of parameters. When tested on a real TBAD case reconstructed from CT, the prediction of FL status was in excellent agreement with the 3-year follow-up CT scan in terms of thrombus location and volume. The improved model also reduced computational time by ∼65%, making it more feasible for clinical use. Weissmann et al. developed a pre-clinical FE model of heart failure with preserved left ventricular ejection fraction (HFpEF) using CMR data and intracardiac pressures from swine heart models subjected to pressure overload. Different HFpEF phenotypes were studied, evaluating changes in mechanical properties. An isotropic change in myocardial passive behavior was observed, the magnitude of which was heavily dependent on the degree of hypertrophy. Moreover, hypertrophy emerged as an initial compensatory response to HFpEF, with myocardial thickening enabling a steady transition in passive properties while maintaining tissue incompressibility.

Kohli et al. showed that CFD analysis also has the potential to predict the outcome of clinical procedures, thereby improving patient safety. Specifically, they assessed the impact of a catheter-based laceration of the anterior mitral leaflet to prevent LV outflow obstruction (LAMPOON) on the hemodynamic outcomes of transcatheter mitral valve replacement (TMVR). In a quantitative comparison with a virtual simulation of TMVR without LAMPOON, the LAMPOON procedure achieved a critical increment in the outflow area that was effective in improving LV outflow hemodynamics, particularly in subjects with a small neo-LV outflow tract.

To tackle the scarcity of clinical measurements, personalized computational simulations can still be performed by appropriately calibrating the model parameters. To this end, Shen et al. satisfactorily reproduced the cerebral hemodynamics in the circle of Willis (CoW) in patients with acute subarachnoid aneurysmal hemorrhage by calibrating the parameters of a block diagram hydraulic model with transcranial Doppler flow velocity measurements of CoW vessels. Tanade et al. focused on the numerical quantification of fractional flow reserve (FFR) in coronary arteries, demonstrating that streamlined models can be defined that minimize the set of required patient-specific inputs while also achieving good agreement with gold standard invasive FFR measurements. The proposed approach offers a flexible framework that can expedite the data collection and simulation pipeline, also enabling the analysis of cases with missing data.

When patient-specific boundary conditions are not available for CFD analysis, Tricarico et al. proposed a pipeline to non-invasively estimate the parameters of three-element Windkessel (WK3) models for the hemodynamic assessment of the aortic arch arteries. A set of normalized WK3 parameters was defined based on ultrasound-derived patient-specific flow rates and intra-operatively measured pressure waveforms. When patient-specific flow rate and pressure waveforms are not available, these normalized parameters can be combined with readily available vessel diameter, brachial blood pressure, and heart rate data to obtain patient-specific WK3 parameters. Bhardwaj et al. developed an *in vitro* anatomical cerebrovascular model to study the mechanism of acute ischemic stroke (AIS). The experimental model was used to benchmark corresponding CFD predictions, which proved to be in relatively close agreement with experimental measurements for both normal and stroke conditions in terms of pressure and flow rate. This represents an important milestone toward validating a computational AIS model, providing additional insights into the dynamics of embolus migration and lodging in the brain, which are difficult aspects to measure *in vivo*.

The final step of a computational workflow involves the post-processing and visualization of relevant data. However, results are often viewed on a 2D display and as static images. To address these limitations, Venn et al. developed a semi-automated workflow for more enhanced and interactive viewing of CFD results in an immersive virtual environment (IVE). The increasing popularity of virtual and augmented reality IVEs is expected to provide more detailed insights from biomedical CFD simulations and also promote greater collaboration between bioengineers and clinicians.

The studies collected in this Research Topic share the common goal of advancing the personalization of cardiovascular computational modeling: model reliability can be improved by combining cardiovascular imaging with clinical data while increasing the usability of computational analysis in real clinical settings by minimizing computational effort.

